# Platelet-derived microparticles serve as an important source of autoantigens and discriminate between levels of disease activity in systemic lupus erythematosus

**DOI:** 10.1186/ar4633

**Published:** 2014-09-18

**Authors:** Eric Boilard, Vincent Bissonnette, Valérie Garceau, Ellie Aghdassi, Nathalie Cloutier, Claudia Beaudoin, Davy Eng, Stacey Morrison, Paul R Fortin

**Affiliations:** 1 Centre de recherche du CHU de Québec, Université Laval, Quebec City, QC, Canada; 2 Toronto Western Research Institute, Toronto, ON, Canada; 3 CHU de Québec - CHUL, Quebec City, QC, Canada

## Background

Immune complexes (IC) are implicated in the pathogenesis of several autoimmune diseases including systemic lupus erythematosus (SLE). In SLE, submicron extracellular vesicles, called microparticles (MP), are thought to serve as an antigenic surface promoting the deposition of immunoglobulins and the formation of MP-associated immune complexes (mpICs). However, the cellular origin of these mpICs is unknown and whether they correlate with disease activity and particular clinical features remains to establish.

## Methods

The concentrations of mpICs in platelet-poor plasma from 193 women with SLE were determined using high-sensitivity flow cytometry. Considering the recently revealed role of platelets in SLE, we further scrutinized the contribution of platelets to mpICs formation. The platelet and nonplatelet MPs and mpICs were tested for association with lupus disease activity, damage, history of previous arterial disease, and the carotid intima-media thickness and plaque area on ultrasound. To assess whether disease activity and damage are associated with levels of MPs and mpICs, univariate and multivariate negative binomial models were built using the SLE disease activity index 2000 (SLEDAI-2K) and the SLICC/ACR damage index (SDI) as outcome variables. In all models, the predictor variable was the level of MPs or mpICs. When necessary, models were adjusted for covariables such as age, disease duration, menopausal status, hypertension, diabetes, anticoagulant or antiplatelet medication, antimalarial medication, prednisone use, smoking status, and ethnicity.

## Results

The clinical characteristics of the 193 women studied were: age (mean (SD)) 46.3 (14.7) years; disease duration 18.5 (12.0) years; ethnicity (% Caucasian) 57%; ever-smoker 34%; menopausal in 55%; hypertensive 30%; diabetic 5%; prescribed anticoagulant or antiplatelet medication 25%; prescribed antimalarial medication 74%; prescribed prednisone 44%. Univariate analyses for activity revealed that platelet-derived mpICs, but not mpICs from other cells, were associated with SLEDAI-2K. In the multivariate model, this association remained significant (*P *= 0.02 for annexin V^+ ^platelet mpICs and *P *= 0.0006 for annexin V^-^platelet mpICs) after adjusting for disease duration, hypertension and currently on prednisone. There was no association between platelet mpICs and SDI.

## Conclusions

Platelet-derived MPs are a major source of autoantigens serving mpIC formation in SLE. Platelet-derived mpICs are associated with lupus disease activity level on the SLEDAI-2K but not with damage. This is the first report of an association between platelet mpICs and clinical marker of activity in SLE and in any autoimmune disease. Platelet mpICs need to be further considered as a possible biomarker of lupus disease activity.

